# In health and unemployment: well-being impacts of joblessness on oneself and partners

**DOI:** 10.1007/s10754-026-09419-9

**Published:** 2026-07-06

**Authors:** Israel Escudero-Castillo, Fco. Javier Mato-Díaz, Ana Rodríguez-Álvarez

**Affiliations:** 1 Department of Applied Economics, University of Burgos, Burgos, Spain; 2 Department of Applied Economics, University of Oviedo, Asturias, Spain; 3 Department of Economics, University of Oviedo, Asturias, Spain

**Keywords:** Unemployment, Psychological well-being, Gender differences, Two-stage least squares regression, Instrumental variables

## Abstract

This paper examines the direct and indirect or vicarious (i.e., experienced through a partner’s unemployment) relationship between unemployment and psychological well-being, shedding more light on why unemployment patterns may differ between women and men. To accomplish this, we tackle the potential endogeneity concern by employing an identification strategy designed to explore the associations between unemployment and mental health while reducing biased results. To our knowledge, this study is the first to employ this strategy to analyse both direct and indirect relationships. We utilize individual and household data from Spain across the years 2006, 2011, and 2017, representing the first study about this topic for this country. The results indicate a detrimental link between unemployment and personal well-being. By sex, a significant association with unemployment was only observable in men, while among women, it became significant only when disaggregated by parental status, specifically among those without children. A similar pattern emerges for the vicarious association: male unemployment is associated with worse psychological well-being among their female partners, especially in couples without children. By contrast, female unemployment is not significantly associated with the psychological well-being of their male partners.

## Introduction

In addition to the possibility of earning a salary, employment is related to a series of functions such as a schedule, shared experience with unfamiliar people, individual goals, personal status and identity as well as physical activity (Jahoda, 1981). This conceptualization expands the value of employment beyond its financial importance and makes it more of an element that provides people with a sense of personal identity which may be tied to the particular work role or the more general social role of breadwinner in a family (Price et al., 1998). The effect of being unemployed therefore manifests itself through the absence of these financial and non-financial functions, thus affecting the mental health of the unemployed person (Janlert & Hammarström, 2009; Paul & Batinic, 2010).

There is abundant literature that focuses on how individual unemployment is related to worse mental health in the person suffering it (Cygan-Rehm et al., 2017; O’Leary et al., 2020; Paul & Moser, 2009). The link between unemployment and mental health or well-being is potentially bidirectional, suggesting that each can influence the other. Researchers have tackled this risk through various methods, ultimately providing evidence consistent with a causal relationship between unemployment and mental health or well-being (Paul & Moser, 2009; Urbanos-Garrido & Lopez-Valcarcel, 2015). Recently, new meta-analyses have provided sound additional evidence about the negative association between unemployment and mental health and subjective well-being (Gedikli et al., 2023) and about the contribution of work to lower psychological distress and higher life satisfaction (Aitken et al., 2023). However, literature has paid less attention to examining the effects of employment status on family members. For people with partners and/or dependent family members, this sense of personal identity, linked to gender roles and family dynamics, may generate spillover effects on family well-being. Both economic and emotional help may be affected by the employment situation and, thus, family well-being may be adversely affected by joblessness.

Our proposal includes a gender perspective, examining differences between men and women in both the direct effects and the vicarious associations of unemployment. The role of children is also considered as a potential contextual or moderating factor in the analyses.

In order to consider the relationship between unemployment and mental health while addressing potential sources of bias, this paper uses an identification strategy consisting of the estimation of a model based on simultaneous equations and using instrumental variables. This methodology is novel in the literature that attempts to estimate the spillover or indirect associations of unemployment.

This study contributes to the literature by highlighting how joblessness may act as a critical juncture in the life course, examining how the disruption caused by unemployment within family dynamics relates not only to the mental health of the unemployed individual but also to that of their partner. To the best of our knowledge, this is the first country-level study that analyses unemployment spillover associations within couples in Spain. Instead of examining only the primary or direct effect of unemployment on the mental health of the individual experiencing it, we also analyse a secondary, or vicarious, association affecting their partner. While the analysis allows for a more robust (causal) interpretation of the relationship at the individual level, the evidence on partner outcomes should be understood as associational. It is particularly appealing to investigate this issue in a country with severe unemployment problems, significant gender differences in the labour market, and a major role played by the institution of the family. Moreover, this work offers suggestive evidence that may help explain why unemployment tends to have a less detrimental effect on women compared to men.

The paper is structured as follows: first, we introduce the related literature in the next section. Then, Sect. 3 is devoted to presenting the analytical method, and Sect. 4 to explaining data sources and variables. Section 5 describes research results and Sect. 6 concludes.

## Literature review

The relationship between unemployment and the psychological well-being (PWB) of individuals has been extensively studied, revealing significant negative impacts on the unemployed (Cygan-Rehm et al., 2017; Eliason & Storrie, 2009; Frijters et al., 2010; Gnambs et al., 2015; O’Leary et al., 2020; Paul & Moser, 2009). PWB is usually approximated from a score obtained in a psychometric test (Cygan-Rehm et al., 2017; Urbanos-Garrido & Lopez-Valcarcel, 2015), levels of alcohol consumption (Jørgensen et al., 2019; Mangot-Sala et al., 2021), overall mortality (Neshat Ghojagh et al., 2024) or incidence of suicide (Milner et al., 2014) among others. Risk of bidirectional causation is tackled through different methods like using panel data (Clark, 2003; Krug & Eberl, 2018), looking at exogenous increases in unemployment (Urbanos-Garrido & Lopez-Valcarcel, 2015) or analysing plant closures (Browning & Heinesen, 2012), among others. Although evidence on adverse causal impact of unemployment is predominant, it is not totally consistent, with some research suggesting that the selection effects of individuals with health issues entering unemployment may contribute to the observed correlation between poor health and unemployment (Schmitz, 2011; Stauder, 2019).

In the existing literature, earlier studies primarily drew upon data from men within the labour market, resulting in a relative scarcity of evidence regarding the effects of unemployment on women (Dew et al., 1991). Upon comparing the available evidence by gender, it becomes apparent that the negative impact of unemployment tends to be stronger for men than for women (van der Meer, 2014). Thus, the detrimental effects of unemployment on mental health particularly affect men, who tend to experience a more significant decline in well-being (Gedikli et al., 2023).

The interpretation of gender differences regarding unemployment effects on well-being may lie on a set of domains that may affect the role configuration of men and women differently: family circumstances, social approval and the centrality of work (Ensminger & Celentano, 1990; van der Meer, 2014). One such feature may come from differences in the opportunity cost of work and unemployment. The unequal distribution of housework between men and women reduces women’s labour market participation and working hours (Samtleben & Müller, 2022). In societies where female work is characterized by relatively poor conditions (lower wages, temporary employment or part-time work) work attachment and links to the labour market may be reduced for women. If women become unemployed, their loss will be less than that of unemployed men and, consequently, the direct effect of unemployment will be lower for women (Gili et al., 2016; Strandh et al., 2013). By contrast, in societies with a similar level of labour market attachment for men and women, there would be no differences in the way in which unemployment affects the mental health of males and females.

Theoretical foundations of the differences between men and women regarding unemployment effects point to the social production function theory, which posits that individuals ultimately seek physical well-being and social validation. Since unemployment impacts both physical well-being and social approval, its effect is substantial. Given that men and women pursue social approval through different avenues, unemployment tends to be more detrimental for men than for women (van der Meer, 2014).

Gender differences regarding unemployment experiences may also depend on family features, specifically the presence of minors. Different social roles in raising children and different levels of investment that men and women make in raising children may cause such differences. This theory has been supported by various studies, some of which highlight the reduced protective role against mental distress typically associated with being employed among mothers, while others suggest a lesser negative impact of joblessness. In terms of the first, Helbig et al. (2006) find that full-time employment is associated with lower rates of psychological distress among fathers but not among mothers when compared with part-time work or unemployment. Plaisier et al. (2008) reach similar conclusions: having a job protects men regardless of whether they have children or not. However, in the case of women it only protects those who are not mothers. Finally, Leupp (2017) concludes that being a mother reduces the protective factor of both full and part-time employment, although the health effects of paid work tend to increase as children grow up.

Regarding the lesser negative impact of joblessness, Russo et al. (2021) find a decreased psychological impact of unemployment on Italian women and relate it to a greater investment on the part of women in their role as caregivers. This is related to the existence of traditional gender stereotypes still common in Italy which associate the role of women with family care work and the role of men with being family breadwinners. In line with this, Heyne and Voßemer (2023) find that women suffer less from unemployment mainly due to non-financial factors such as weaker labour market attachment and traditional gender roles. Finally, Artazcoz et al. (2004) looked at the Autonomous Community of Catalonia in Spain. They found that, among women, unemployment was associated with poor mental health but only among childless women, and especially among those who work in high level occupations.

The importance of the family for understanding the differential effects of unemployment on men and women suggests that effects may extend beyond the individual, affecting other family members. (Beck, 2016; Dew et al., 1991; Howe et al., 2004; Maitoza, 2019; Nikolova & Nikolaev, 2021; Pedersen et al., 2005; Song et al., 2011; Ström, 2003).

Nikolova and Ayhan (2019) worked with a sample of German couples in which one of the partners had become unemployed due to company closure, thereby addressing potential sources of bias arising from the possibility that individuals lose their jobs due to poor mental health (two-way causation). The authors found that the unemployment of one of the partners had a negative influence on the life satisfaction of the other, estimating that this vicarious effect accounts for 25% of the effect caused by unemployment itself. The results, moreover, were insensitive to household income, which demonstrates the non-pecuniary value of employment and the effect of its social value. Analysing data from the German Socioeconomic Panel, Bünnings et al. (2017) found that the fear of job loss negatively impacts spouses’ mental well-being, with a stronger effect in single-income households. In another study based on a sample of people residing in Germany who had lost their jobs due to firm closure, Marcus (2013) concluded that this vicarious effect of unemployment is as important as the primary one.

Using data from the British Household Panel Survey, Mendolia (2014) finds evidence of the impact of male unemployment on men’s own PWB and on the wives of those men who have experienced job loss. The author also points to non-pecuniary factors such as low self-esteem and individual perceived role as contributing explanatory factors.

Some authors state that this transmission could depend on the employment situation of the recipient (Clark, 2003). Thus, if the partner of the unemployed person is also unemployed, the vicarious effect will be less detrimental in terms of psychological distress when compared with having employed partners. The author explains this counter intuitive result using the concept of social norm. Hence, the unemployment of the member receiving the effect will moderate the social norm deviation of the sender’s joblessness and, therefore, the vicarious effect of the lack of employment. The situation in this regard is ambiguous, since Marcus (2013) not only fails to confirm this result but also presents findings to the contrary. However, what appears certain is that there are indications showing that unemployment affects not only the individual experiencing it, but also their partners.

According to Westman et al. (2004), from a theoretical point of view, gender also plays a moderating role on the vicarious effect of unemployment: the effect of male unemployment on the mental health of their female partners is greater than the effect of female unemployment on that of their male partners. This gender difference has been demonstrated at European level (van der Meer, 2014) and in countries such as Germany (Esche, 2020; Knabe et al., 2016; Voßemer et al., 2024), the United Kingdom (Cochrane & Stopes-Roe, 1981) or Australia (Bubonya et al., 2017).

This phenomenon could be explained through Becker’s theory of specialization (Becker, 1991), according to which the unit of decision is not the individual, but the family itself. Households can use their time by selling it on the labour market, using it in domestic production, or consuming goods and services. According to Becker, each adult member of the household will focus on those activities that maximize family well-being depending on the benefit they provide. Thus, the different wages and working conditions that affect men and women within the same family will influence their specialization. For this reason, if women’s wages are lower and their working conditions worse, the opportunity costs that women will have to bear due to staying out of the labour market will be lower than those for men. Thus, the way in which families will tend to maximize their well-being may be conditioned by gender stereotyped division of production, and therefore the vicarious effect of women’s unemployment will be lower than that of men.

The objective of this research is to contrast whether these theoretical concepts are supported within the context of Spain. The evidence to justify our focus on Spain comes from the distinctive conditions that characterize this nation regarding labour market problems, gender disparities, and the role of the family. First, regarding labour market problems, despite having reached a historical record of over 22.2 million registered workers in mid-2025, Spain remains a world leader in unemployment. According to the most recent data from Eurostat (January 2026), the unemployment rate of 9.8% is 4% points above the European Union (EU) average of 5.8%. This unfortunate leadership in unemployment is a traditional feature of the Spanish economy. Differences in unemployment rates are strikingly high, with Spain standing out as one of the highest in the EU, only surpassed by Finland (10.0%). Frequent transitions between employment and unemployment situations contribute to the deepening segmentation of the Spanish labour market, which may be putting the mental health of part of the population at risk (Benach et al., 2023; Escudero-Castillo et al., 2023).

Second, regarding gender disparities, the Spanish labour market has usually been characterised by significant gender differences. The Spanish Labour Force Survey (September to December 2025) reported an unemployment rate of women reaching 11.24%, while that of men was 8.76%. Temporary employment is also higher for women than for men (17.6 vs. 12.8%). Additionally, women had a significantly higher rate of part-time employment (21.5% vs. 7%). The Statistical Office also provided data on the gender wage gap, revealing that in 2023 women’s median (21,177.99) salary was 84.4% of men’s (25,088.03). Since 2010, female labour force participation has increased notably in Spain, accompanied by gradual shifts in gender norms. Yet, while more women are active in the labour market, they continue to face structural disadvantages such as occupational segregation, job precarity, and unequal responsibility for caregiving and domestic work (Hupkau & Ruiz-Valenzuela, 2022; Lozano & Rentería, 2019). These persistent inequalities shape both labour market outcomes and the mental health effects of unemployment, particularly for mothers (Guner et al., 2014). The COVID-19 lockdown further deepened these gender disparities: although men slightly increased their participation in home production, women experienced a sharper increase in unpaid work and a smaller reduction in paid work hours, leading to a widening of the total work gap between genders (Farré et al., 2022).

Third, in Spain the family is an institution of reference both in economic and social terms, as seen by the key role played by family members both before and during the Great Recession, providing emotional and financial support (Alonso, 2012; Mínguez, 2017). In Spanish families, gender disparities abound, as the task of looking after dependent family members is largely borne by women (Farré & González, 2019). Thus, labour market situations and the gender of an individual can feed each other in shaping well-being, with the vicarious effects of unemployment potentially contributing to these dynamics. All of this therefore makes analysing Spain and including family variables in this research relevant and timely.

## Methods

We analyse the effect of unemployment on mental health considering individual and family characteristics. As already explained, the unemployment variable (U) may have a causal effect on PWB, but these variables may also be spuriously related due to unobserved factors. To address the issue of bidirectional causality, a two-stage model (2SLS) is proposed. A 2SLS model consists of a two-stage estimation technique. In the first stage, endogenous variables are predicted using instrumental variables along with other exogenous variables. In the second stage, the main dependent variable is regressed on the predicted values obtained in the first stage, substituting the endogenous variable with its predicted value.

Since the hypothesized endogenous variable (being unemployed or not) is a dummy variable, the first stage of the model should consist of a binary regression model. Thus, in the second stage, it would involve a regression where the PWB is regressed by “replacing a nonlinear function of an endogenous explanatory variable with the same nonlinear function of fitted values from a first-stage estimation” (Wooldridge, 2010, p. 236). This second estimation is what is known as a “forbidden regression” (Angrist & Pischke, 2009, p. 142; Wooldridge, 2010, p. 236) and could lead to biased and inconsistent estimates, violating the necessary assumptions for valid instrumental variable estimation. Ultimately, the problem in the estimation arises because only OLS estimation guarantees that the residuals from the first stage are uncorrelated with the fitted values and covariates, something that is not assured when using fitted values from a nonlinear model (Angrist & Pischke, 2009).

An alternative to the forbidden regression when using a binary response model in the first stage is to use the nonlinear fitted values as the instrumental variable instead of directly substituting the fitted values in the second stage. In this approach, the nonfitted values refer to the predictions based on the nonlinear model, but instead of using the fitted probabilities from the first stage directly as an explanatory variable in the second stage, they are used as an instrument for the endogenous variable (Angrist & Pischke, 2009; Wooldridge, 2010; Xu, 2021). Although this methodology has been successfully applied in previous studies (Adams et al., 2009), to our knowledge, the present analysis is among the first applications of this fitted-probability instrumental-variable approach addressing the link between employment status, psychological well-being and partner associations using data from the Spanish National Health Survey on couples.

This approach starts from Eq. (1) where PWB is a function of several individual and family variables (vector *X*) and unemployment (*U*):

1 $$\mathrm{PWB} = \delta_{1} + \delta_{2}\,X + \delta_{3}\,U + u$$

Where δ’s are parameters to be estimated. We assume that Cov(*X*, *u*) = 0 but u is thought to be correlated with *U*, that is to say, Cov(*U*, *u*) ≠ 0. If we also assume that *z* is a valid instrument for *U*, we can estimate the following binary response model by maximum likelihood in a first stage:

2 $$U = \beta_{1} + \beta_{2}\,X + \beta_{3}\,z +\varepsilon$$

Where *ε* is the error term in the reduced form (2), and *z* must fulfil the following conditions: Cov(*z*, *u*) = 0; and *β*_3_ ≠ 0 which means that *z* is partially correlated with *U* once the other exogenous variables *X* have been netted out. When *z* satisfies both conditions, then it is said to be an instrumental variable candidate for *U*. In a second stage, the estimated fitted probability of unemployment obtained by (2) is used as instrument. Using (1) and (2) and rearranging we get:

3 $$\mathrm{PWB}\:=\alpha_{1}+\alpha_{2}\,X+\alpha_{3}\, z+ v$$

Where *v* = *δ*_3_*ε* + *u*; *α*_2_ = *δ*_2_ + *δ*_3_*β*_2_; and *α*_3_ =* δ*_3_*β*_3_. Under our assumptions, *v* is uncorrelated with all explanatory variables in Eq. (3) and so the model consistently estimates the *α*_j_ parameters.

## Data

This research uses cross-sectional data from the Spanish National Health Survey (NHS; Ministry of Health, Social Services and Equality). The data corresponds to three waves (2006, 2011 and 2017) and to both the individual and household databases. This survey is made up of people residing in family households within Spanish territory. The large samples used, the diversity of health variables analysed, and the inclusion of variables related to social characteristics make it an excellent reference survey for examining the relationship between socioeconomic and health determinants. This survey also benefits from incorporating the 12-item General Health Questionnaire (GHQ-12; 12-item version). This is the most frequently used instrument in Europe (Cygan-Rehm et al., 2017; Paul & Batinic, 2010; Robone et al., 2011) for measuring the psychological state of unemployed people.

All these characteristics make the NHS an ideal database for carrying out this research. Initially, the total sample consisted of 73,574 individuals. However, many participants had to be excluded for the following reasons. Firstly, because of missing values in the GHQ-12 scores, education level, partner’s employment status, marital status, and household income. Secondly, because they did not belong to the economically active population, defined as employed or unemployed but seeking work. Thirdly, to evaluate the influence of the gender regime, individuals with same-sex partners were excluded. Finally, those who did not live with their partners or whose partners were not in the labour market were also removed from the sample. After applying all these criteria, the final sample comprised 16,311 individuals, of whom 13,806 were workers and 2,505 were unemployed.

While the sample includes only individuals who are economically active—either employed or unemployed and actively seeking work—the dataset does not distinguish between voluntary and involuntary unemployment. This limits our ability to assess differences in mental health effects based on the nature of job loss. Previous research suggests that involuntary unemployment tends to have more negative consequences, and we acknowledge this as a limitation in interpreting our findings.

The research required merging the three individual databases available from the NHS, which contain both health and work information. Also, in order to carry out the analysis of vicarious association of unemployment and other family factors, the household databases from the three waves^1^ were also merged. A final merge using the available identification number of households permits the obtaining of a complete database with individual and family information.

Regarding mental health and the use of the GHQ-12, to operationalise the proposed dependent variable and following the correction instructions of the questionnaire manual (Lobo & Muñoz, 1996), we have dichotomized the GHQ-12 scores, applying the GHQ bimodal scoring method (0-01-1).^2^ Specifically, each of the 12 items was recoded as 0 (for original Likert responses 0 and 1, indicating absence of distress) or 1 (for original Likert responses 2 and 3, indicating presence of distress). The final dependent variable was calculated as the sum of these 12 dichotomized items, resulting in a score ranging from 0 to 12, where higher scores reflect worse PWB.

In our model, unemployment is introduced as the independent variable and PWB as the dependent variable (Eq. 1 in the Methods Section). Partner’s unemployment, sex, age, years of schooling, household income, the number of minors in the household, marital status, the province where the person lives and the GDP by region and year are used as explanatory variables. Following the approach outlined in Sect. 3, two previous probit models^3^ are estimated with unemployment as the dependent variable and sex, partner’s unemployment, age, years of schooling, and the number of minors in the household as independent variables (Eq. 2). The predicted probabilities from these probit models are used as instrumental variables for individuals’ unemployment.

Therefore, in addition to the previous independent variables, one of the probit models includes the provincial unemployment rate, disaggregated by sex and by the year of the NHS edition, as well as the square of this rate. The other probit model includes the labour force participation rate, also disaggregated by province, sex, and year, along with the square of this rate. As pointed out by Alonso-Borrego and Carrasco (2017), Gathergood (2013), and Álvarez Llorente (2002), these variables exploit information about local labour market conditions, which is useful for predicting the employment status of individuals. This approach is consistent with the use of aggregate labour market indicators as sources of exogenous variation in individual employment outcomes in related studies.

Under this approach, it is assumed that this provincial information on the unemployment rate and labour force participation rate is uncorrelated with the error term. More specifically, the identifying assumption is that, conditional on individual characteristics and province fixed effects, these variables affect PWB primarily through their impact on individual employment status.

While provincial labour market conditions could in principle influence PWB through contextual channels like public service provision or social cohesion, several elements mitigate these concerns. First, province fixed effects absorb time-invariant regional characteristics that may be correlated with both labour market conditions and wellbeing. Second, identification relies on within-province variation over time in labour market conditions, as well as differences across sex, which reduces the likelihood that the instruments capture persistent contextual factors.

Moreover, labour force participation rates reflect broader patterns of labour market attachment and are less likely to be directly linked to adverse economic conditions, making a direct effect on PWB less likely than in the case of unemployment rates. Nevertheless, this assumption should be interpreted with caution.

We requested data on the provinces in which each person resides from the Spanish Ministry of Health, the provincial unemployment and labour force participation rates being calculated for each of the three periods and separately for men and women, based on the information collected in each of the three NHS editions. Table 1 presents the specification of the variables included in the analysis, and Table 2 provides descriptive statistics.

**Table 1:** Variables included in the analysis

Variable	Rank
Unemployment	1 if the person is unemployed; 0 if the person is working
Partner’s unemployment status	1 if the partner is unemployed; 0 if the partner is working
GHQ-12 score (PWB)	Scored from 0 to 12, with higher scores indicating lower Psychological Well-Being (PWB).
Sex	1 if the person is a man; 0 if the person is a woman
Age	Continuous variable
Years of Schooling	Continuous variable
Household income	1 if the household in which the respondent lives has low income (≤ 800 euros); 2 if the household has lower-middle income (801–1,300 euros); 3 if the household has middle income (1,301 to < 2,200 euros); and 4 if the household has upper-middle or high income (≥ 2,200 euros).
Nº minors	Number of minors in household. Continuous variable
Marital status	1 if the person is single; 2 if is the person is married; 3 if the person is widowed; 4 if the person is divorced or separated
GDP by region and year	Continuous variable
Provincial unemployment rate (IV)	Continuous variable
Provincial labour force participation rate (IV)	Continuous variable

Source: own elaboration

**Table 2:** The composition of employed and unemployed people

	People with work		Unemployed people		Total	
Sample size (N)	13,806		2,505		16,311	
Partner’ employment situation (%)						
Employed	88.87		74.53		86.67	
Unemployed	11.13		25.47		13.33	
GHQ-12 scores (average)						
	1.08		1.94		1.21	
Distribution by sex (%)						
Male	47.02		35.57		45.26	
Female	52.98		64.43		54.74	
Age (average)						
	42.06		41.83		42.02	
Years of schooling (average)						
	13.82		12.24		13.58	
Household income (average in thousands of euros)						
	3.14		2.37		3.02	
Minors in the household (average)						
	0.86		0.88		0.87	
Marital status (%)						
Single	11.02		11.90		11.15	
Married	86.98		85.47		86.75	
Widowed	0.24		0.24		0.24	
Divorced/separated	1.76		2.40		1.86	
GDP by region and year (average)						
		81,400,000	84,000,000		81,800,000	
Provincial unemployment rate (average)						
		14.28	18.84		14.98	
Provincial labour force participation rate (average)						
		58.45	56.14		58.10	

Source: own elaboration based on the 2006, 2011 and 2017 Spanish National Health Survey.

Finally, Table 3 shows the descriptive (unadjusted) GHQ-12 average by gender, and the employment situation of the individual and their partner. In all cases, people with the worst mental health are those who are unemployed and whose partners are also unemployed. On the other hand, those with the lowest mental health risk are those who are employed and whose partners are also employed. Situations in which one partner is employed while the other is unemployed fall in an intermediate range between these two extremes. It should be noted that women exhibit higher average GHQ-12 scores than men in almost all combinations of their own and their partner’s employment status (employed vs. unemployed), suggesting that baseline gender differences in reported mental health are not solely attributable to labour market factors. However, there are important differences by gender in the association between employment status and mental health. First, among men, employment seems to be more strongly related to better mental health, as the gap between employed and unemployed individuals is larger than among women, regardless of the partner’s labour situation. In other words, for men, employment appears to have a stronger association with better mental health than for women.

**Table 3:** PWB by own and partner employment status

	EMPLOYED PARTNER		UNEMPLOYED PARTNER	
	Mean (SD)	*N*	Mean (SD)	*N*
TOTAL SAMPLE				
Employed	1.05 (1.95)	12,270	1.27 (2.26)	1,536
Unemployed	1.82 (2.72)	1,867	2.29 (3.01)	638
MEN				
Employed	0.80 (1.68)	5,497	1.00 (2.07)	995
Unemployed	1.99 (2.80)	575	2.14 (2.87)	316
WOMEN				
Employed	1.25 (2.12)	6,773	1.77 (2.50)	541
Unemployed	1.75 (2.69)	1,292	2.44 (3.13)	322

Source: own elaboration.

GHQ-12 scored using the bimodal method (0–12). Values are means with standard deviations in parentheses. Table reports descriptive (unadjusted) statistics. N denotes number of observations in each cell.

Secondly, the association with worse mental health of having an unemployed partner appears to be greater for women, regardless of their own employment status. Thus, the average is higher when the unemployed partner is a man (1.77 vs. 1 and 2.44 vs. 2.14).

Finally, it is worth noting that women who are employed but have unemployed partners have almost the same mean as women who are unemployed with employed partners. This would suggest that, for these women, the association with better mental health of their own employment is similar to that associated with their partner’s employment, which is not the case for men. For men, their own unemployment is much more strongly associated with a higher risk of psychological distress than their partner’s unemployment. These descriptive differences should be interpreted with caution, as they do not account for observable or unobservable confounding factors, which are addressed in the multivariate analysis below.

## Results

In Tables 4, 5 and 6, estimates from Ordinary Least Squares (OLS) and Two-Stages Least Squares (2SLS) are presented for the complete sample (Model 1), as well as separately for men (Model 2) and women (Model 3). In addition, models are presented for men and women depending on the presence of children in the household (Model 4 for women with children, Model 5 for women without children, Model 6 for men with children, and Model 7 for men without children). Table 5 reports the first-stage results of the 2SLS estimation. As previously described, an estimation that does not account for the reciprocal causality between PWB and unemployment status could lead to biases in the estimates. Therefore, from here on, only the results presented in Table 6 will be explained. All models in Table 6 are estimated using robust standard errors clustered at the province-sex-year level, which corresponds to the level of variation of the instruments.^4^

**Table 4:** Ordinary least square regressions

	MOD 1 General sample	MOD 2 Men	MOD 3 Women	MOD 4 Women with children	MOD 5 Women without children	MOD 6 Men with children	MOD 7 Men without children
Unemployment (ref: no)	0.6101***	0.9453***	0.4128***	0.2282**	0.6711***	0.9256***	0.9409***
	(0.0610)	(0.1033)	(0.0761)	(0.0918)	(0.1292)	(0.1364)	(0.1562)
Partner’s unemployment (ref: no)	0.1942***	0.0763	0.3643***	0.5829***	0.1002	0.1655*	−0.0219
	(0.0582)	(0.0691)	(0.1009)	(0.1378)	(0.1482)	(0.0916)	(0.1062)
Sex (ref: woman)	−0.3917***						
	(0.0334)						
Age	0.0041**	0.0040	0.0027	−0.0000	0.0026	0.0048	0.0029
	(0.0020)	(0.0027)	(0.0030)	(0.0053)	(0.0039)	(0.0045)	(0.0036)
Years of schooling	−0.0121***	−0.0013	−0.0224***	−0.0157**	−0.0311***	−0.0030	−0.0003
	(0.0037)	(0.0047)	(0.0053)	(0.0070)	(0.0083)	(0.0062)	(0.0073)
Household income	−0.2379***	−0.2330***	−0.2103***	−0.1838***	−0.2360***	−0.1754***	−0.2890***
	(0.0284)	(0.0401)	(0.0408)	(0.0518)	(0.0640)	(0.0501)	(0.0648)
Minors in household	−0.0075	0.0064	−0.0160				
	(0.0194)	(0.0263)	(0.0279)				
Marital Status (ref: single)							
Married	−0.2130***	−0.0636	−0.3210***	−0.3106**	−0.3620***	−0.0441	−0.0542
	(0.0592)	(0.0772)	(0.0871)	(0.1253)	(0.1220)	(0.1183)	(0.1062)
Widowed	0.6792	−0.3113	0.9239	−0.4954	1.6651**	−1.0687***	−0.0223
	(0.4877)	(0.3462)	(0.6163)	(0.7384)	(0.7977)	(0.2054)	(0.4066)
Divorced/separated	0.3003*	0.4631**	0.1740	0.1416	0.1770	0.6711*	0.3091
	(0.1617)	(0.2283)	(0.2273)	(0.3241)	(0.3134)	(0.3667)	(0.2748)
GDP by region and year	−0.0000	−0.0000	−0.0000	0.0000	−0.0000	−0.0000	0.0000
	(0.0000)	(0.0000)	(0.0000)	(0.0000)	(0.0000)	(0.0000)	(0.0000)
Constant	2.2410***	1.8039***	2.2283***	1.6742***	2.9143***	2.0059***	1.5372***
	(0.2779)	(0.3901)	(0.3978)	(0.5026)	(0.6203)	(0.5256)	(0.5859)
Observations	16,311	7,383	8,928	4,908	4,020	4,182	3,201
R-squared	0.0567	0.0677	0.0467	0.0487	0.0630	0.0681	0.0841

***Statistical significance: 1%; ** Statistical significance: 5%; *Statistical significance: 10%

Dummies Provinces are included in the estimation

Robust standard errors in parentheses

**Table 5:** First-stage linear regressions with instrumental variables

	MOD 1 General sample	MOD 2 Men	MOD 3 Women	MOD 4 Women with children	MOD 5 Women without children	MOD 6 Men with children	MOD 7 Men without children
Unemployment probability	1.0943***	1.1067***	1.0421***	1.2193***	0.7677***	1.1525***	1.0455***
	(0.0621)	(0.0644)	(0.0960)	(0.1234)	(0.1262)	(0.0777)	(0.1010)
Labour force participation probability	−0.0857	−0.5686**	0.1515	0.4685	−0.0240	−0.4426*	−0.9259**
	(0.1609)	(0.2328)	(0.3539)	(0.4690)	(0.4265)	(0.2657)	(0.4135)
Partner’s unemployment (ref: no)	−0.1198***	−0.0538*	−0.1566**	−0.2761***	−0.0456	−0.0677*	−0.0115
	(0.0228)	(0.0322)	(0.0568)	(0.0724)	(0.0650)	(0.0358)	(0.0506)
Sex (ref: woman)	0.0091						
	(0.0124)						
Age	0.0013***	0.0023***	0.0004	−0.0010	0.0008	0.0022**	0.0021***
	(0.0004)	(0.0004)	(0.0006)	(0.0011)	(0.0007)	(0.0007)	(0.0006)
Years of schooling	0.0071***	0.0054***	0.0075**	0.0121***	0.0042	0.0064***	0.0030
	(0.0012)	(0.0016)	(0.0028)	(0.0035)	(0.0033)	(0.0019)	(0.0024)
Household income	−0.1607***	−0.1724***	−0.1485***	−0.1606***	−0.1334***	−0.1617***	−0.1824***
	(0.0071)	(0.0084)	(0.0107)	(0.0131)	(0.0125)	(0.0097)	(0.0117)
Minors in household	0.0037	−0.0061*	0.0122*				
	(0.0035)	(0.0034)	(0.0054)				
Marital status							
Married	−0.0126	−0.0232**	−0.0008	−0.0229	0.0159	−0.0426**	−0.0080
	(0.0091)	(0.0117)	(0.0134)	(0.0217)	(0.0166)	(0.0198)	(0.0175)
Widowed	−0.0555	−0.1720***	−0.0102	−0.1111	0.0376	−0.1360**	−0.1787***
	(0.0464)	(0.0440)	(0.0581)	(0.0824)	(0.0762)	(0.0575)	(0.0594)
Divorced/separated	−0.0092	−0.0214	0.0012	−0.0256	0.0284	−0.0374	−0.0147
	(0.0247)	(0.0279)	(0.0389)	(0.0509)	(0.0547)	(0.0437)	(0.0391)
GDP by region and year	0.0000***	0.0000***	0.0000	0.0000	0.0000	0.0000***	0.0000***
	(0.0000)	(0.0000)	(0.0000)	(0.0000)	(0.0000)	(0.0000)	(0.0000)

***Statistical significance: 1%; ** Statistical significance: 5%; *Statistical significance: 10%

Dummies Provinces are included in the estimation

Cluster-robust standard errors in parentheses

**Table 6:** Second-stage linear regressions with instrumental variables

	MOD 1 General sample	MOD 2 Men	MOD 3 Women	MOD 4 Women with children	MOD 5 Women without children	MOD 6 Men with children	MOD 7 Men without children
Unemployment (ref: no)	1.4373***	1.8735***	0.7669	−0.1043	2.5382**	1.2401**	3.1165***
	(0.4119)	(0.5216)	(0.6482)	(0.6479)	(1.0850)	(0.4903)	(0.6974)
Partner’s unemployment (ref: no)	0.1737***	0.0644	0.3462***	0.5885***	−0.0569	0.1588*	−0.0277
	(0.0614)	(0.0644)	(0.1117)	(0.1324)	(0.1819)	(0.0900)	(0.1131)
Sex (ref: woman)	−0.3380***						
	(0.0592)						
Age	0.0032	0.0019	0.0026	−0.0006	0.0013	0.0043	−0.0020
	(0.0025)	(0.0033)	(0.0036)	(0.0065)	(0.0040)	(0.0042)	(0.0046)
Years of schooling	−0.0116***	−0.0040	−0.0212***	−0.0168**	−0.0265***	−0.0039	−0.0068
	(0.0035)	(0.0049)	(0.0053)	(0.0077)	(0.0082)	(0.0061)	(0.0068)
Household income	−0.1047	−0.0706	−0.1590	−0.2362*	0.0071	−0.1240	0.1141
	(0.0762)	(0.1010)	(0.1111)	(0.1232)	(0.1603)	(0.0990)	(0.1454)
Minors in household	−0.0137	0.0103	−0.0220				
	(0.0186)	(0.0228)	(0.0312)				
Marital Status (ref: single)							
Married	−0.2022***	−0.0461	−0.3195***	−0.3195**	−0.3883***	−0.0323	−0.0429
	(0.0693)	(0.0719)	(0.1042)	(0.1330)	(0.1178)	(0.1215)	(0.1204)
Widowed	0.7280	−0.1393	0.9297	−0.5410	1.5910**	−1.0229***	0.3991
	(0.4501)	(0.3184)	(0.5846)	(0.7789)	(0.7245)	(0.3662)	(0.3829)
Divorced/separated	0.3099**	0.4829**	0.1760	0.1289	0.1265	0.6815*	0.3442
	(0.1435)	(0.2156)	(0.1897)	(0.3046)	(0.2702)	(0.3927)	(0.2419)
GDP by region and year	−0.0000	−0.0000	−0.0000	0.0000	−0.0000	−0.0000**	−0.0000
	(0.0000)	(0.0000)	(0.0000)	(0.0000)	(0.0000)	(0.0000)	(0.0000)
Underidentification test (Kleibergen-Paap rk LM statistic)	85.053***	44.886***	41.472***	42.031***	21.793***	41.730***	34.461***
Weak identification test Cragg-Donald Wald F statistic^5^ Kleibergen-Paap rk Wald F statistic	276.339 172.660	160.349 147.517	98.508 74.440	77.927 69.266	23.376 21.705	115.610 110.885	49.120 54.734
Overidentification test of all instruments (Hansen J statistic)	0.239	0.861	0.927	0.140	0.842	0.253	0.388
Observations	16,311	7,383	8,928	4,908	4,020	4,182	3,201
R-squared	0.0252	0.0296	0.0245	0.0206	−0.0380	0.0436	−0.0575

***Statistical significance: 1%; ** Statistical significance: 5%; *Statistical significance: 10%

Dummies Provinces are included in the estimation

Cluster-robust standard errors in parentheses

As explained in the Methods section, in each of the 2SLS models, the estimated probabilities of unemployment from two probit models were used as instrumental variables. Among other independent variables, one probit model included the provincial unemployment rate by year and sex along with its square, while the other included the provincial labour force participation rate by year and sex and its square.

Regarding the complete model (Model 1 in Table 6) the result of the endogeneity test (chi-square = 4.436; *p* = 0.0352) allows rejecting the null hypothesis of no endogeneity in unemployment. Additionally, the positive sign of the coefficient indicates the presence of unobservable variables that are increasing the probability of suffering a psychological problem and that, at the same time, said unobservable variables are also increasing the probability that the person is unemployed. Essentially, the significant result of the test seems to be related to the endogeneity of the unemployment variable, justifying the choice of simultaneous equations and instrumental variables methodology.

Moreover, the results allow us to assess whether the necessary conditions for using a variable as an instrument are satisfied. The first of these conditions is relevance, meaning that the instrument must be correlated with the endogenous variable. The Kleibergen–Paap test evaluates whether the model is identified, that is, whether the instruments provide sufficient information about the endogenous regressors. In this case, the Kleibergen–Paap rk LM statistic is highly significant in all models (*p* < 0.001), indicating that there are no issues with underidentification.

The other condition for using an instrumental variable is that it must not be correlated with the error term of the model, which implies that the effect of the instrumental variable on the dependent variable occurs only through the endogenous variable. This is known as the exogeneity condition. This condition can be assessed using Hansen’s overidentification test. The null hypothesis of this test is that the instrument is exogenous. In all models presented in Table 6, the Hansen test does not reject the overidentifying restrictions, although this result alone cannot establish the exclusion restriction.

To further ensure the reliability of these findings, all models were re-estimated incorporating sampling weights (elevation factors). These results, which are highly consistent with the baseline unweighted estimates, can be consulted in Table 8 of the Appendix. This stability across specifications provides additional evidence of the robustness of the reported coefficients.

As for the complete sample (Model 1), the absence of employment is related with a higher risk of mental health problems suffered by the jobless person. Similarly, partner unemployment is associated with PWB: people who live with an unemployed partner show decreased psychological health compared to people who live with employed people. Specifically, unemployed individuals are estimated to have, *ceteris paribus*, more than 1.4 points higher score compared to employed individuals. Additionally, individuals living with an unemployed person are estimated to have a 0.17 point higher score compared to those living with an employed person. The years of schooling are significantly related to PWB. Thus, each year of schooling reduces the GHQ-12 score more than 0.01 points. Finally, a single person has better mental health when compared with separated/divorced people, but worse one when compared with married people.

Regarding gender, women are more likely than men to suffer psychological problems. Specifically, being a woman is associated with an increase, on average, the GHQ-12 score by 0.34 points. This result is consistent with other research carried out in Spain (Rocha et al., 2010) which demonstrates that being a woman, regardless of employment status, is related with a higher probability of obtaining a score corresponding to psychological risk.

In order to delve into the differential effect of unemployment by gender, Models 2 and 3 present the result for men and for women respectively. In Model 2, the coefficient of the unemployment variable is positive and significant. Specifically, for men, unemployment is associated with an increase in the GHQ-12 score of more than 1.8. For women, the employment situation is not statistically significant. Therefore, this result is in line with research suggesting that unemployment situation is associated with differential effects between men and women (Gili et al., 2016). Regarding the unemployment vicarious association, having an unemployed partner is associated with an increase in the GHQ-12 score of more than 0.3 points but only in the case of women, that is, only when the unemployed partner is male. In the case of men, the unemployment of their female partners is not associated with any increase in the GHQ-12 score. Finally, years of schooling, and marital status show similar behaviour as in Model 1 —regarding the first variable only within the women’s subsample. In the case of men subsample, only marital status variable is significant.

With the objective of clarifying the difference between men and women regarding the unemployment (direct and vicarious) coefficient, additional models are proposed. Given the aforementioned literature on the moderating role of motherhood on unemployment and the evidence regarding the growth of gender gaps following motherhood (De Quinto et al., 2021), we have created four more models, two for women with and without children (Model 4 and Model 5) and other two for men, again, with and without children (Model 6 and Model 7). The hypothesis put forward is that the worsening of labour conditions for women, especially after becoming mothers, may be related to a lesser association between unemployment and their PWB. This may be more salient in couples where women have taken on a secondary role in the labour market due to motherhood. Therefore, the male vicarious association of unemployment with women’s PWB will be greater in couples with children than in those without. This hypothesis is based on evidence demonstrating that the gender gap exacerbates after the onset of motherhood and that the association between employment and PWB is conditioned by the existence or not of certain congenial working conditions (Barnay, 2016; Bildt & Michélsen, 2002).

To further support this interpretation, we provide descriptive evidence on gender differences in employment conditions, household income, and caregiving roles. Women in our sample face more precarious employment situations, with higher rates of temporary contracts (16.2% vs. 15.25% for men) and verbal-only agreements (4.1% vs. 0.97%). They are also far more likely to work reduced hours (7.14% vs. 0.77%), indicating a weaker attachment to the labour market. In terms of household income, women are underrepresented in the highest income bracket (24.89% vs. 28.81%), suggesting lower household reliance on their earnings. Finally, caregiving responsibilities are unequally distributed: 14.99% of women provide regular care to someone elderly or chronically ill, compared to only 4.19% of men. These patterns are consistent with evidence suggesting that, in a somewhat traditional country like Spain, childcare responsibilities still fall mainly on women.^6^ Plausible contextual explanations for our results could relate to the idea that lower opportunity cost, reduced household dependence, and greater caregiving roles may help explain why joblessness appears as less detrimental to mothers’ psychological well-being, although these mechanisms are not directly tested in our analysis.

**Graph 1:**
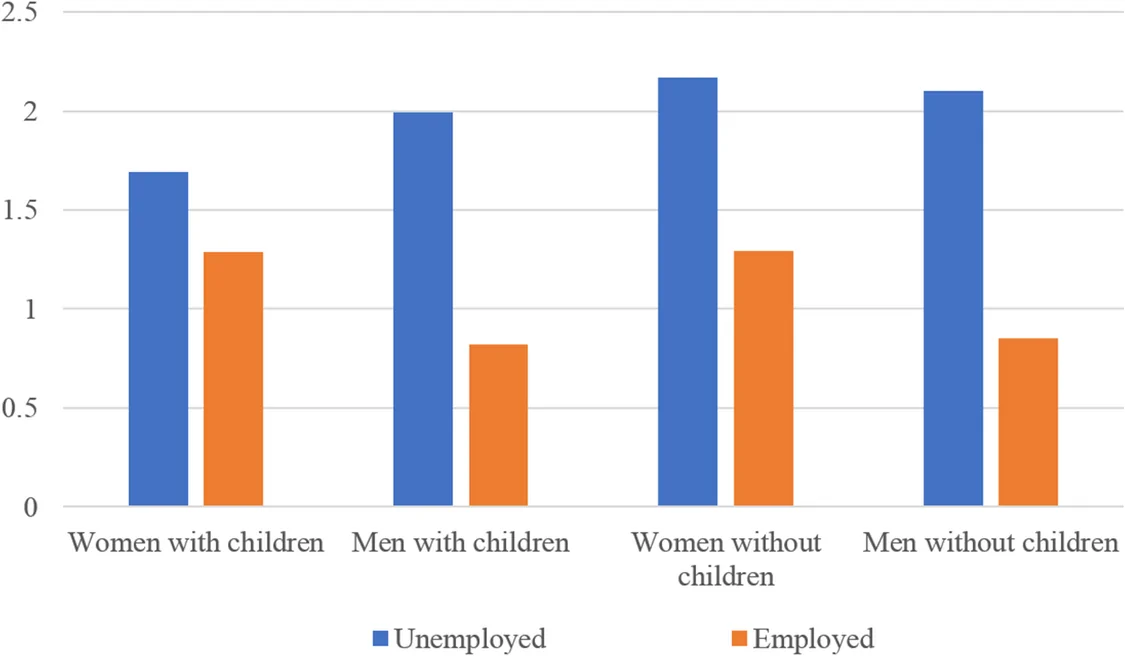
Mean Distress Risk scores of employed and unemployed depending on gender and the presence of children. Source: own elaboration. Notes: GHQ-12 (0–12, bimodal scoring)

Before commenting on the results, we present Graph 1, which contains the representation of the mean GHQ-12 (0–12) scores of employed and unemployed men and women depending on gender and the presence of children. Indeed, both unemployed men and women (in blue) have a higher mean score on the GHQ-12 scale that employed (in orange), indicating poorer PWB. However, this score also appears to vary depending on the presence of children. Unemployed men, and very especially women, have a lower score (which is related to better PWB) if they have children. Among employed individuals, there are no differences depending on the presence of children in the household.

Models 4 and 5 display the results of estimating the previous models for women, distinguishing between those with children and those without. Thus, among women with children, unemployment remains statistically non-significant. However, for childless women, unemployment becomes significant and has a positive effect. In concrete terms, for women without children, unemployment is associated with an increase in the GHQ-12 score of more than 2.5 points. These results should be interpreted as differences across subsamples rather than a direct effect of motherhood. Thus, estimated coefficients show that when the effect of unemployment on women with and without children is taken into account, the negative association of unemployment is more clearly observed among women without children.

For the Spanish population, the threshold for considering an individual at risk of experiencing a PWB problem is three (Artazcoz et al., 2004; Urbanos-Garrido & Lopez-Valcarcel, 2015). The estimated coefficients for the general sample (Model 1) show that, even starting from an initial situation with no risk (a GHQ-12 score of zero), unemployment increases the score to around half of the critical threshold.

This risk is particularly pronounced among men, especially those without children. In this group, unemployment is associated with GHQ-12 levels that may exceed the threshold for psychological risk, thus classifying individuals as at risk. Among women, the effect is observed only in those without children, who also reach a level close to the risk threshold.

As for the vicarious association of male unemployment with women’s PWB, it is only observed when the female partner has been a mother (Model 4). In concrete terms, the man’s unemployment is associated with an increase of almost 0.6 points in his partner’s GHQ-12 score. If the woman has not been a mother (Model 5), the unemployment of the man is not associated with worse mental health in women.

Among men, the effect of unemployment appears regardless of fatherhood status. Although this effect is greater among men without children, it is also observed among those with children. Being unemployed increases GHQ-12 scores by 1.2 points for men with children, while for men without children, it increases their score by 3 points. Again, as described for the full male sample, the vicarious association of unemployment cannot be demonstrated; that is, when the unemployed individual is a woman, no significant relationship is observed.

These findings align with some research that has concluded that the greater effect of unemployment on men (as compared to women) may be moderated by parenthood. For example, Helbig et al. (2006) conclude that full-time employment (compared to unemployment) is associated with lower rates of disorders among fathers, but not among mothers. In this same vein, Plaisier et al. (2008) conclude that employment protects women, but only those without children. For a Spanish sample, Artazcoz et al. (2004) conclude that unemployment had a greater effect on men, and that this effect could be due to the presence of children in the household; in fact, among women with children at home, unemployment failed to be associated with worse mental health. Finally, Russo et al. (2021) determine that having a child softens the effect of unemployment on PWB, and that this effect is greater for women than for men, based on a sample from Italy.

To conclude, other variables also show significant results and follow the pattern observed in Models 2 and 3. With regard to education level, more years of schooling are linked to a lower risk of poor PWB, but only in the case of women. Similarly, among women, and specifically those who are mothers, income exhibits a protective effect on psychological well-being. Being married (compared to being single) provides greater protection for women—both with and without children, with very similar coefficients—than for men, for whom the coefficients are not statistically significant. For men, being single (compared to widowed) and being divorced (compared to single) are associated with worse PWB among men with children.

Before concluding, it is worth noting some limitations related to sample selection. In particular, several exclusion criteria were applied, including the removal of individuals with missing information, those not belonging to the economically active population, and those not living with a partner or whose partner was not in the labour market. As a result, the final sample may not be fully representative of the broader population. These restrictions may limit the external validity of the findings, particularly for groups not captured in the analysis. However, they are necessary to ensure consistency in the definition of key variables and to allow for the analysis of within-couple dynamics.

## Conclusions

The objective of this research is to analyse the effect that an individual’s employment situation has on mental health, taking into account their personal and family characteristics (such as their partner’s employment status or child-rearing responsibilities). To carry out this analysis, six official databases from Spain were used. The distinctive characteristics of the Spanish labour market make this study very relevant. Spain stands out as the EU member state with the highest unemployment rate and a high gender wage gap, which is consistent with the possibility that the outcomes of unemployment may differ between men and women. Also, Spain is considered to be a country with labour market segmentation very marked by gender, and one in which domestic and care work is predominantly feminized. These patterns lead us to expect that gender differences may play a significant role in the way in which each person interprets their work situation.

Simultaneous equations models allow us to account for the potential endogeneity of unemployment. Moreover, to test the robustness of these results, various models have been estimated such as logistic regression models, linear regression models with endogenous treatment effects (with and without sample weights) or bivariate probit models. In all cases, the conclusions reached are consistent. The results obtained indicate that, indeed, there are unobservable variables affecting both the probability that the person is unemployed and the probability of obtaining a score corresponding with higher psychological risk. Results can be summarized in three parts.

Firstly, the estimated models allow us to provide evidence consistent with the relationship between unemployment situation and worse PWB in Spain. Thus, unemployment situation (compared to employment) significantly increases the GHQ-12 by 1.4 points in the general sample and by 1.8 in men.

Secondly, due to the lack of significant effect of the unemployment variable with the subsample of women, the analysis was repeated by creating four different subsamples: two composed of women with and without children, and two composed of men with and without children. The findings regarding the direct effect of unemployment differ depending on the presence of children in the subsample households: among women with children, as in the general sample of women, it was not possible to demonstrate an impact of individual unemployment on PWB. However, this direct effect of unemployment is statistically significant only among women without children.

Although the gender mechanisms are not directly tested, these results are compatible with Becker’s theory, according to which the labour market penalises women who have decided to be mothers. This penalty may decrease the opportunity cost of not working, such that joblessness is not as painful for women as it is for men. This effect is not observed among women in general, but it is present in the subsample of women living with children.

Perhaps the penalty suffered by women when they become mothers is not limited only in terms of salary. According to Plaisier et al. (2008) this effect could also be related to the role that society attributes to men and women as workers. Thus, women may be less influenced than men by the non-economic benefits of employment, that is, the primitive latent functions of employment (Jahoda, 1981), such as self-esteem and self-realization. Thus, the role of mother could be overshadowing the role of worker. It is expected that this effect will be more significant the more the role of women is associated with the functions of caring for children or housework.

Thirdly, the inclusion of the partner’s employment situation provides evidence consistent with the existence of a vicarious association of unemployment. It is observed in the general sample and when the unemployed person is a man. However, female unemployment does not appear to be associated with psychological harm to their male partners. Perhaps due to the disadvantageous situation of women in the Spanish labour market when compared with men the protective capacity of employed female partners is less significant than that of male partners. As observed in the sex-specific models (Models 2 and 3), when taking into account the presence of children in the household, the vicarious association of unemployment is only evident when the unemployed person is a man and, moreover, in couples with children. Female unemployment is not associated with PWB among their male partners, and male unemployment is also not associated with PWB among their female partners when there are no children in the household.

Additionally, it seems that the presence of children is associated with a stronger role assigned to men as wage earners, as evidenced by an increase in the vicarious association (Model 4 vs. Model 3). Finally, regarding other variables, years of schooling, household income and marital status are also related to PWB.

All of the above allows us to suggest that female employment in Spain has a lower protective power, in terms of PWB, not only for women but also for their male partners. Thus, women’s employment appears to be less strongly associated with PWB compared to men’s employment, and therefore, women’s unemployment condition is less strongly associated with PWB for both themselves and their partners than when the unemployed person is a man. Furthermore, it is the disadvantage of women in the job market, especially after motherhood, that could be explaining these differences.

Despite the contributions of this paper, some limitations should be noted. First, the analysis is based on cross-sectional data rather than panel data, which limits the ability to track changes in individuals’ psychological well-being and labour situation over time. Second, although individuals’ labour situation is instrumented and thus potential reverse causality is addressed, it was not possible to use instrumental variables for partners’ labour situation. This limitation prevents a fully causal assessment of the vicarious effects of unemployment within couples. Therefore, partner effects should be interpreted as associational. Third, our findings provide evidence from the Spanish context, where high unemployment, gendered labour-market segmentation, and family-based care responsibilities may shape the association between unemployment and psychological well-being differently for men and women. Thus, external validity would require further comparative work.

This research concludes pointing out that the inclusion of family variables (employment status of the partner or child rearing responsibilities) is necessary in order to obtain unbiased results when studying the effects of employment situations on mental health. Given the high cost of mental health problems (OECD/European Union, 2018), attention to the relationship between employment situation and mental health, as well as attention to mental health in the workplace, are now considered priorities in government agendas.

## Annex

### General health questionnaire-12

1. Have you recently been able to concentrate on whatever you’re doing?
2. Have you recently lost much sleep over worry?
3. Have you recently felt that you were playing a useful part in things?
4. Have you recently felt capable of making decisions about things?
5. Have you recently felt constantly under strain?
6. Have you recently felt you couldn’t overcome your difficulties?
7. Have you recently been able to enjoy your normal day-to-day activities?
8. Have you recently been able to face up to problems?
9. Have you recently been feeling unhappy or depressed?
10. Have you recently been losing confidence in yourself?
11. Have you recently been thinking of yourself as a worthless person?
12. Have you recently been feeling reasonably happy, all things considered?

For each of the 12 items, the possibilities of response are:

- More so than usual.................... 1
- About the same as usual........... 2
- Less so than usual..................... 3
- Much less than usual................. 4

**Table 7:** Probit models of unemployment

VARIABLES	Probit with provincial unemployment rate as IV	Probit with provincial labour force participation rate as IV
Provincial unemployment rate	0.0714***	
	(0.0072)	
Squared provincial unemployment rate	−0.0009***	
	(0.0002)	
Provincial labour force participation rate		0.0709***
		(0.0190)
Squared provincial labour force participation rate		−0.0006***
		(0.0002)
Sex (ref: women)	−0.2367***	−0.2826***
	(0.0266)	(0.0876)
Partner’s unemployment (ref: no)	0.4244***	0.5180***
	(0.0336)	(0.0332)
Age	−0.0034**	−0.0011
	(0.0015)	(0.0014)
Years of schooling	−0.0512***	−0.0362***
	(0.0029)	(0.0026)
Minors	0.0213	0.0149
	(0.0145)	(0.0143)
Constant	−1.3317***	−2.8985***
	(0.1817)	(0.5684)
Observations	16,311	16,311

Robust standard errors in parentheses

Dummies Provinces are included in the estimation

*** *p* < 0.01, ** *p* < 0.05, * *p* < 0.1

**Table 8:** Survey-weighted second-stage linear instrumental-variable regressions

	MOD 1 General sample	MOD 2 Men	MOD 3 Women	MOD 4 Women with children	MOD 5 Women without children	MOD 6 Men with children	MOD 7 Men without children
Unemployment (ref: no)	1.6374***	1.9629***	0.4229	−0.9258	3.0099**	1.1265***	3.8824***
	(0.4137)	(0.3356)	(0.7688)	(0.6379)	(1.2329)	(0.3423)	(0.6073)
Partner’s unemployment (ref: no)	0.2459***	0.1297	0.4356***	0.6847***	−0.0288	0.3836***	−0.1083
	(0.0739)	(0.0911)	(0.1255)	(0.1250)	(0.2367)	(0.1232)	(0.1771)
Sex (ref: woman)	−0.3318***						
	(0.0604)						
Age	0.0022	−0.0008	0.0049	0.0047	−0.0022	0.0113	−0.0069
	(0.0032)	(0.0043)	(0.0048)	(0.0085)	(0.0064)	(0.0084)	(0.0061)
Years of schooling	−0.0135***	−0.0027	−0.0295***	−0.0275***	−0.0316**	−0.0038	−0.0050
	(0.0046)	(0.0061)	(0.0068)	(0.0100)	(0.0125)	(0.0075)	(0.0085)
Household income	−0.0830	−0.0445	−0.2318*	−0.4037***	0.1060	−0.1961**	0.3087**
	(0.0702)	(0.0714)	(0.1250)	(0.1414)	(0.1990)	(0.0916)	(0.1326)
Minors in household	−0.0175	0.0311	−0.0479				
	(0.0271)	(0.0296)	(0.0545)				
Marital Status (ref: single)							
Married	−0.0949	0.1185	−0.3283**	−0.5064***	−0.1680	0.2475**	0.0510
	(0.0933)	(0.0766)	(0.1474)	(0.1795)	(0.1591)	(0.1106)	(0.1836)
Widowed	0.9142	0.1031	0.9109	−1.7047***	2.2766**	−1.2742	0.6750*
	(0.7187)	(0.4004)	(0.9333)	(0.3428)	(1.1174)	(0.8918)	(0.3738)
Divorced/separated	0.3964*	0.7605**	0.0660	−0.0088	0.0743	1.1823**	0.4463
	(0.2331)	(0.3701)	(0.2443)	(0.3451)	(0.3774)	(0.5948)	(0.3007)
GDP by region and year	−0.0000	0.0000***	−0.0000***	−0.0000***	−0.0000***	0.0000***	0.0000
	(0.0000)	(0.0000)	(0.0000)	(0.0000)	(0.0000)	(0.0000)	(0.0000)
Constant	2.5076**	−0.1894	5.1516***	5.7333***	5.2262***	−0.0005	−0.3211
	(1.0751)	(0.4235)	(0.5708)	(0.8786)	(0.9246)	(0.5546)	(0.6256)
Observations	16,311	7,383	8,928	4,908	4,020	4,182	3,201
R-squared	0.0446	0.0656	0.0617	0.0380		0.1004	

***Statistical significance: 1%; ** Statistical significance: 5%; *Statistical significance: 10%

Dummies Provinces are included in the estimation

Survey-robust standard errors in parentheses

## Footnotes

1 The Spanish National Health Survey has three questionnaires and three different databases. One at the individual level, in which the information about the selected person is contained; another covering the characteristics and composition of the household of the chosen person and, finally, another about the minors residing in the home.

2 The complete General Health Questionnaire-12 is attached as an Annex to this paper.

3 The results of the probit models are provided in the Annex of this article (Table 7).

4 All specifications include province fixed effects using the *partial()* option of the *ivreg2* command in the Stata program. This removes the fixed effects from all other variables prior to estimation, ensuring that the coefficients of the remaining regressors are unaffected. The option is especially useful when calculating clustered standard errors, as it helps maintain a full-rank covariance matrix and avoids numerical problems that can arise with many fixed effects or singleton dummies.

5 For a model with one endogenous regressor and two instruments, the Stock–Yogo (2005) weak ID testcritical values (at the 5% significance level) are: 19.93 (10% maximal IV size), 11.59 (15%), 8.75 (20%), and 7.25 (25%).

6 For Spain, the most recent data available regarding the distribution of domestic and care tasks by gender dates back to 2016 (Eurofound’s European Quality of Life Survey,2016). The average number of hours a week that men dedicate to the care and education of their children in Spain is 23 and the average dedicated by women 38. Similarly, the number of hours that women dedicate to housework (20) is also higher than the number of hours that men dedicate (11). 2016). The average number of hours a week that men dedicate to the care and education of their children in Spain is 23 and the average dedicated by women 38. Similarly, the number of hours that women dedicate to housework (20) is also higher than the number of hours that men dedicate (11).
