# Correction to: In health and unemployment: well-being impacts of joblessness on oneself and partners

**DOI:** 10.1007/s10754-026-09422-0

**Published:** 2026-09-24

**Authors:** Israel Escudero-Castillo, Fco. Javier Mato-Díaz, Ana Rodríguez-Álvarez

**Affiliations:** 1 Department of Applied Economics, University of Burgos, Burgos, Spain; 2 Department of Applied Economics, University of Oviedo, Asturias, Spain; 3 Department of Economics, University of Oviedo, Asturias, Spain

**Correction to: Int J Health Econ Manag. (2026) 26:15**

**https://doi.org/10.1007/s10754-026-09419-9**

In Table 1 of this article, the entry “Partner’ employment situation” were incorrect and should have been “Partner’s unemployment status.

In Table 5, 6, and 8 of this article, the entry “Sex (ref: man)” were incorrect and should have been “Sex (ref: woman)”. 

The original article has been corrected.

